# Management of flu-like syndrome with cetirizine in patients with relapsing-remitting multiple sclerosis during therapy with interferon beta: Results of a randomized, cross-over, placebo-controlled pilot study

**DOI:** 10.1371/journal.pone.0165415

**Published:** 2017-07-07

**Authors:** Doriana Landi, Maria Albanese, Fabio Buttari, Fabrizia Monteleone, Laura Boffa, Silvia Rossi, Caterina Motta, Elisa Puma, Diego Centonze

**Affiliations:** 1 Multiple Sclerosis Clinical and Research Unit, Department of Systems Medicine, Tor Vergata University, Rome, Italy; 2 IRCCS Istituto Neurologico Mediterraneo (INM) Neuromed, Pozzilli (Is), Italy; 3 Neuroimmunology and Neuromuscular Diseases Unit, IRCCS Fondazione Istituto Neurologico Carlo Besta, Milan, Italy; 4 Biogen Italy, Medical Department, Milan, Italy; Cardiff University, UNITED KINGDOM

## Abstract

**Background:**

Flu-like syndrome (FLS) is a common adverse event experienced by patients with relapsing-remitting multiple sclerosis (RRMS) treated with interferon beta (IFNβ). FLS can lead to poor treatment adherence and early IFNβ discontinuation. The involvement of interleukin-6 (IL-6) in the occurrence of FLS has been suggested. We hypothesized that cetirizine, a second-generation histamine H1 receptor antagonist able to reduce the levels of IL-6, might improve IFNβ-induced FLS.

**Methods:**

We conducted a pilot, cross-over, randomized, placebo-controlled, double-blind study to evaluate the efficacy of cetirizine 10 mg added after each IFNβ injection to the standard of care for FLS (acetaminophen or nonsteroidal anti-inflammatory drugs) on FLS in patients with RRMS treated with IFNβ. Patients were randomized to two treatment sequences: 1) 4-week treatment with placebo added to the standard treatment for FLS, followed by 4-week treatment with cetirizine added to the standard of care, and 2) first addition of cetirizine, then of placebo. The primary efficacy endpoint was the mean change of FLS severity [11-point visual analog scale (VAS)] after 4 weeks of treatment within each sequence.

**Results:**

Forty-five patients (71.1% female, mean age 39.1 years, mean time from RRMS diagnosis 5.8 years) were randomized to treatment sequences 1 and 2. The differences between cetirizine and placebo in the intensity of FLS were not statistically significant: total mean VAS scores at 4 hours from IFNβ injection were 3.57 and 3.42 for cetirizine and placebo, respectively (difference –0.15; 95% confidence interval: from –0.74 to 0.44; p = 0.6029). The two treatments were similar also with regard to other efficacy measures considered and to the safety/tolerability profile.

**Conclusions:**

The addition of cetirizine to the standard of care for IFNβ-induced FLS in patients with RRMS does not seem to provide significant benefits compared with placebo. Further effort is required to understand the mechanisms underlying IFNβ-induced FLS.

**Trial registration:**

EudraCT 2013-001055-12.

## Introduction

Interferon beta (IFNβ), used in the management of multiple sclerosis for more than two decades, is among the currently recommended first-line disease-modifying therapies for patients with relapsing-remitting multiple sclerosis (RRMS) [1]. IFNβ has generally proven to be a well-tolerated drug, with the most frequent adverse events being reactions at the injection site and the flu-like syndrome (FLS) [2]. The FLS, which affects approximately 75% of patients injecting IFNβ, includes symptoms like fever, chills, muscle pain, weakness, and headache [3]. These symptoms usually occur 3–6 hours after IFNβ injection and resolve within 24 hours. Their incidence typically declines during the first 3 months of therapy, but in some patients FLS may persist or recur resulting in poor adherence to therapy and early discontinuation of IFNβ [3,4]. Current strategies to prevent or reduce FLS include injecting IFNβ in the evening (to “sleep through” the symptoms), gradual up-titration of IFNβ, prophylaxis with acetaminophen or non-steroidal anti-inflammatory drugs (NSAIDs) and, for the most severe cases, low-dose oral corticosteroids [3,5–9].

The mechanisms underlying IFNβ-related FLS are largely unknown. The scant evidence available suggests the involvement of interleukin-6 (IL-6), a pro-inflammatory cytokine, as the expression of IL-6 appears to be induced by IFNβ [10–12]. In the earlier studies investigating the mechanism of IFNβ-related FLS, higher levels of IL-6 were shown to correlate with increased severity of FLS [10,12], while the improvement of FLS observed in RRMS patients following the addition of low-dose steroids to acetaminophen at the onset of IFNβ therapy was found to be associated with a decrease in IL-6 induction [11]. IL-6 may therefore be an interesting target in the development of more effective strategies for the management of IFNβ-related FLS.

Cetirizine is a second-generation, long-acting, selective histamine H1 receptor antagonist, with proven efficacy and a favorable safety and tolerability profile in the management of allergic disorders in adults, adolescents and children [13]. Cetirizine counteracts the allergic response to antigenic stimuli through several mechanisms including anti-allergic, anti-histaminic, and anti-inflammatory effects [13]. In particular, studies *in vitro* and *in vivo* have shown that cetirizine reduces eosinophil migration induced by inflammatory mediators, diminishes the expression of adhesion molecules associated with eosinophil migration and the adhesion of eosinophils to epithelial cells, and inhibits the expression of various pro-inflammatory molecules, including IL-6 [13].

We hypothesized that, thanks to its multiple effects on inflammatory responses and its ability to decrease IL-6 secretion, cetirizine given before each IFNβ injection might improve or prevent FLS. We tested this hypothesis in a pilot, randomized, placebo controlled, cross-over trial involving patients with RRMS in therapy with IFNβ (Flu-LIGHT study, **Flu L**ike **I**nhibition **G**iving anti-**H**istamine **T**herapy).

## Methods

### Study design

**Fig 1:**
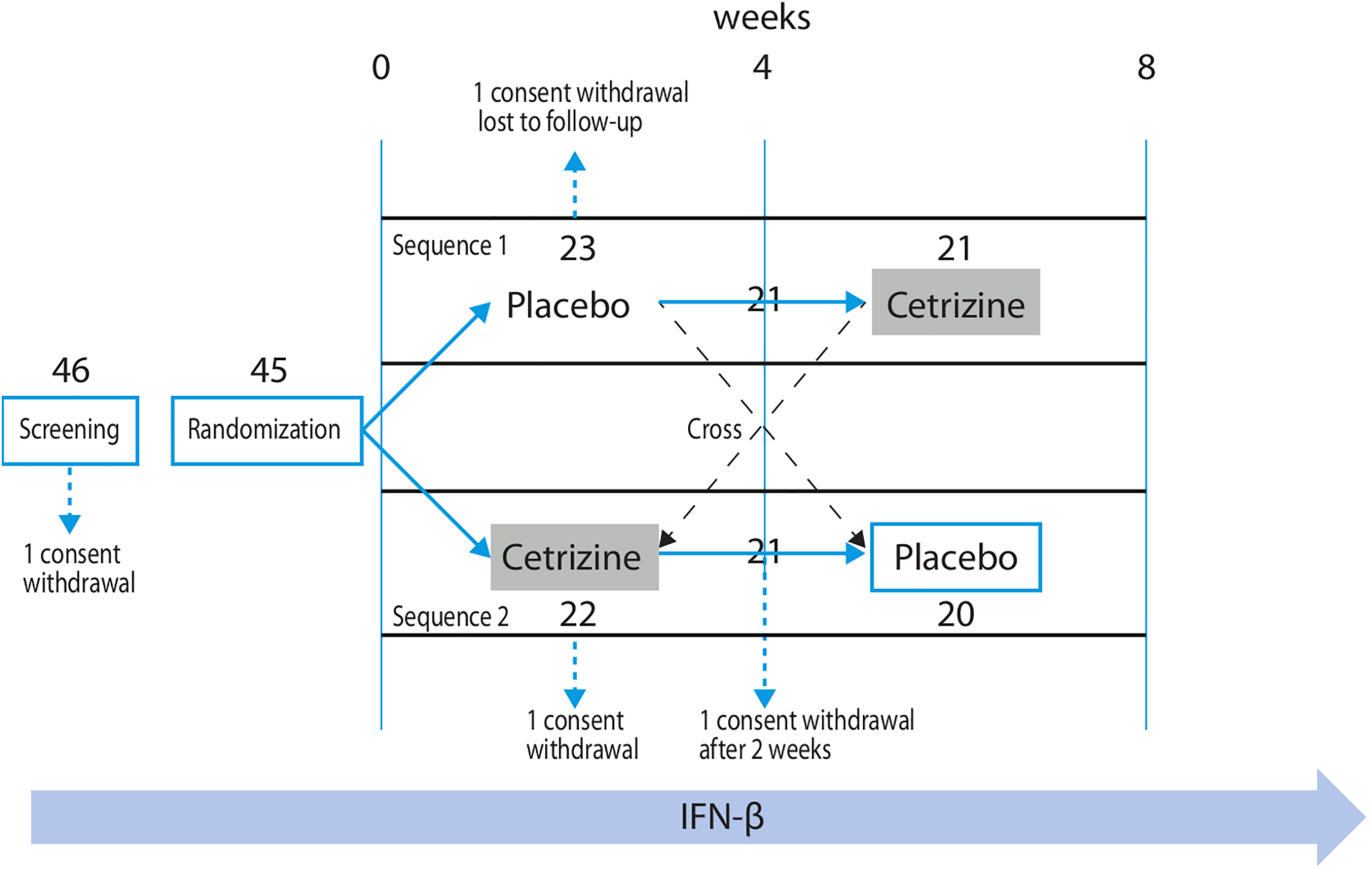
CONSORT diagram of patient flow throughout the study. IFNβ = interferon-β.

This was a pilot, cross-over, randomized, placebo-controlled, double-blind, phase IIIb study Fig 1; S1 File and S1 Checklist. Enrollment was carried out from October 2013 to April 2014 at a MS center in Italy (Policlinico Universitario Tor Vergata, Rome) to evaluate the efficacy of cetirizine on FLS symptoms in patients with RRMS treated with IFNβ (study registered with the EudraCT number 2013-001055-12). There are no ongoing or related studies requiring further registration. The study was conducted in accordance with the International Conference on Harmonization Guidelines for Good Clinical Practice, the Declaration of Helsinki, and the regulations of the Italian Medicines Agency (AIFA) and European Clinical Trials. Each patient provided a written informed consent. Ethical approval for the study was granted on May 28^th^, 2013 by the Comitato Etico Indipendente del ‘Policlinico di Roma Tor Vergata’. The first patient was enrolled on Oct 13^th^, 2013 and the last follow-up was performed on June 15^th^, 2014.

### Patients

The study enrolled patients with RRMS treated with IFNβ for at least 3 months and affected by FLS despite receiving the standard of therapy for FLS [ie, acetaminophen or non-steroidal anti-inflammatory drugs (NSAIDs)]. Patients were using following one of the IFNβ products: subcutaneous IFNβ-1b (Betaferon^®^, Bayer), subcutaneous IFNβ-1a (Rebif^®^, Merck Serono), and intramuscular IFNβ-1a (Avonex^®^, Biogen). Inclusion criteria were: subjects of both sexes; age ≥18 years and ≤55 years; diagnosis of RRMS; therapy with IFNβ for at least 3 months; negative pregnancy test performed no more than 30 days from the baseline visit; FLS-score (FLS-S) ≥2 despite standard therapy for FLS; ability to provide written informed consent for participation in the study; absence of clinically relevant conditions or situations which in the opinion of the investigator would interfere with the study evaluation or with the participation in the study; use of effective birth control methods, or condition of menopause for at least 6 months. Exclusion criteria were: subjects (male or female) potentially fertile not using contraception; pregnant or breastfeeding women; intolerance or known contraindications to the use of cetirizine; hereditary problems of galactose intolerance, Lapp lactase deficiency, glucose-galactose malabsorption; contemporary participation in other studies.

### Treatment allocation

Patients were randomized to two treatment sequences: sequence 1, 4-week treatment with standard therapy for FLS (acetaminophen or NSAIDs) plus placebo, followed by 4-week treatment with standard therapy plus cetirizine 10 mg; and sequence 2, the opposite of sequence 1 (cetirizine in the first period and placebo in the second period). Allocation to the treatment sequence was generated by the Contract Research Organization (CRO) utilizing the completely automated nQuery program to guarantee anonymity and confidentiality. Each subject was assigned a "Unique Trial Subject Code" (UTSC) and a "Personal Identification Code" (PIC). At randomization, each PIC was assigned a randomization number (RN), a sequential number between 01 and 40, which corresponded to the number on the medicinal product used by the subject.

During the study each subject was therefore identified using the UTSC, the PIC and the CodRand (Randomization Code, consisting of PIC+RN), which was recorded on the questionnaire and patient diary. The randomization list was balanced every four subjects and prepared by the CRO and held in a password-protected file by the person in charge of packaging at the production site, with a copy archived by the CRO. Only the Scientific Director of the CRO and the person controlling packaging at the production site had access to the randomization list until unblinding was authorized by the Pharmacovigilance Service of Biogen Idec Italy Srl. The investigator and staff of the trial center, the Sponsor and the CRO were blind to the sequence throughout the duration of the study.

Cetirizine 10 mg and placebo (both as oral capsule formulations) were to be taken 1 hour before each injection of IFNβ. The dosage of standard therapy for FLS was maintained as stable as possible. Dose and frequency of IFNβ therapy were also stable.

### Efficacy evaluation

Patient assessment visits were scheduled at the following time points: at baseline (enrollment in the study and randomization); at 4 weeks (end of first period of treatment); and at 8 weeks (end of second period of treatment). The primary efficacy endpoint was the mean change of FLS severity from before IFNβ injection to 4 hours after injection, as assessed by patients on a visual analog scale (VAS) after 4 weeks of therapy within each sequence. Secondary efficacy endpoints were: the mean change in severity of FLS (as assessed using the VAS and the symptom score of FLS, FLS-S) from before IFNβ injection to 4–6 hours and 12–15 hours after injection, over the entire study period; the proportion of patients with a decrease of ≥2 FLS-S points compared to the value before IFNβ injection (responders); and the incidence of FLS (defined as the proportion of patients with an increase ≥2 of FLS-S compared with the value before IFNβ injection) 4–6 hours after injection and 12–15 hours after injection during the entire study period. FLS severity was assessed by the patients on a VAS of 10 cm, where 0 indicated no discomfort due to the FLS, and 10 maximum discomfort imaginable due to the FLS. To get the FLS-S score, patients rated the presence and intensity of muscle pain, chills and weakness. Each symptom was evaluated separately, according to the following scale: 0 = absent; 1 = mild, did not interfere with daily activities; 2 = moderate, enough to interfere with daily activities; 3 = severe, required bed rest. Furthermore, the body temperature was recorded to determine the presence of fever using the following scale: 0: <37.3°C; 1: ≥37.3°C and <37.8°C; 2: ≥37.8°C and <38.4°C; 3: ≥38.4°C. Scores of each symptom were added together to get the FLS-S, which thus ranged from 0 to 12. An increase in the total score ≥2 points compared to the pre-injection score was considered positive for the presence of FLS. Patients entered the VAS and FLS-S scores measured 1 hour before IFNβ injection, 4–6 hours following injection, and 12–15 hours following injection in their diaries, for each IFNβ injection over 8 weeks. Any change in the dose of analgesics was also recorded in the diary.

### Safety evaluation

Safety and tolerability of cetirizine were monitored during the entire study period. All adverse events and serious adverse events were recorded. In particular, the most common adverse reaction related to the administration of cetirizine (sleepiness) was assessed using the validated Italian translation of the Epworth Sleepiness Scale (ESS), at the baseline visit and at the visits at 4 and 8 weeks [14,15]. The ESS score ranges from 0 to 24, with scores >10 indicating excessive daytime sleepiness.

### Sample size calculation

It was hypothesized that, after four hours from administration of IFNβ, the mean difference (d) of the VAS score between the two treatments was equal to or greater than 0.50, where d was expressed in standard units. Choosing α = 0.05 and β = 0.20 (power = 80%), 34 patients had to be enrolled and treated sequentially in a cross-over design, as reported in the randomization list. Taking into account the possibility of a 15–20% drop-out rate, the final calculated sample consisted of 40 subjects in the two sequences.

### Statistical analysis

Data were analyzed by descriptive statistics. The efficacy analysis was performed on the Intention To Treat (ITT) population (i.e., all patients who received at least one dose of cetirizine or one dose of placebo and had, for both products, at least one evaluation before and after taking the drug; patients who did not have at least these four assessments were not evaluated for efficacy). During this study no major protocol violations/deviations occurred, so the ITT and Per Protocol (PP) populations coincided. It was therefore not necessary to perform additional analysis to check for any discrepancies.

To assess the primary efficacy endpoint, an analysis of variance (ANOVA) with repeated measures was performed, in accordance with a cross-over design. The values considered were the VAS scores collected 4 hours from the last IFNβ injection, after 4 weeks of treatment, according to the treatment sequences 1 (placebo/cetirizine) and 2 (cetirizine/placebo). All analyses of the secondary efficacy endpoints were also performed on the ITT population. The mean change in FLS severity was evaluated with an analysis of variance (ANOVA) with repeated measures, similarly to the analysis performed for the primary endpoint. The values considered were VAS scores collected after 4–6 and after 12–15 hours following the last IFNβ injection, after each week of treatment according to the treatment sequences 1 or 2. The same analysis was carried out considering the FLS-S score. The proportions of responders (patients with a reduction in FLS-S ≥2 points) were analyzed with methods appropriate for a cross-over design [16]. The FLS incidence defined as an increase of ≥2 points in the FLS-S from before IFNβ injection to 4–6 hours and to 12–15 hours following IFNβ injection was analyzed as done for the proportion of responders. The safety population included all randomized subjects who received at least one dose of cetirizine or placebo.

## Results

Patient disposition is shown in Fig 1. Overall, 46 patients were screened and enrolled. One patient withdrew consent before randomization. Out of the 45 randomized patients, 2 patients withdrew consent and 1 patient was lost to follow-up. The last patient left the study in June 2014.

Demographic and baseline characteristics are summarized in Table 1. Patients were predominantly female (71.1%). Their mean age was 39.1 years and the mean time from diagnosis of MS was 5.8 years. Subcutaneous IFNβ-1a 44 μg was the most frequently used IFNβ product (68.9%) followed by subcutaneous IFNβ-1b (17.8%), subcutaneous IFNβ-1a 22 μg (6.7%) and intramuscular IFNβ-1b (6.7%).

**Table 1:** Demographic and baseline characteristics (N = 45).

Sex, n (%)		
	Female	32 (71.1)
	Male	13 (28.9)
Age, year		39.1 (9.1)
Height, cm		167.7 (8.4)
Weight, kg		69.1 (13.5)
BMI, kg/m^2^		24.5 (4.2)
Body surface area, m^2^		1.8 (0.2)
EDSS		1.4 (1.1)
Time to diagnosis, year		5.8 (5.0)
IFNß therapy, n (%)		
	IFNß-1a IM	3 (6.7)
	IFNß-1a SC 22	3 (6.7)
	IFNß-1a SC 44	31 (68.9)
	IFNß-1b	8 (17.8)

Unless otherwise indicated, data are reported as mean values (± standard deviation)

BMI = body mass index

EDSS = Expanded Disability Status Scale

IFNβ = Interferon-β

**Fig 2:**
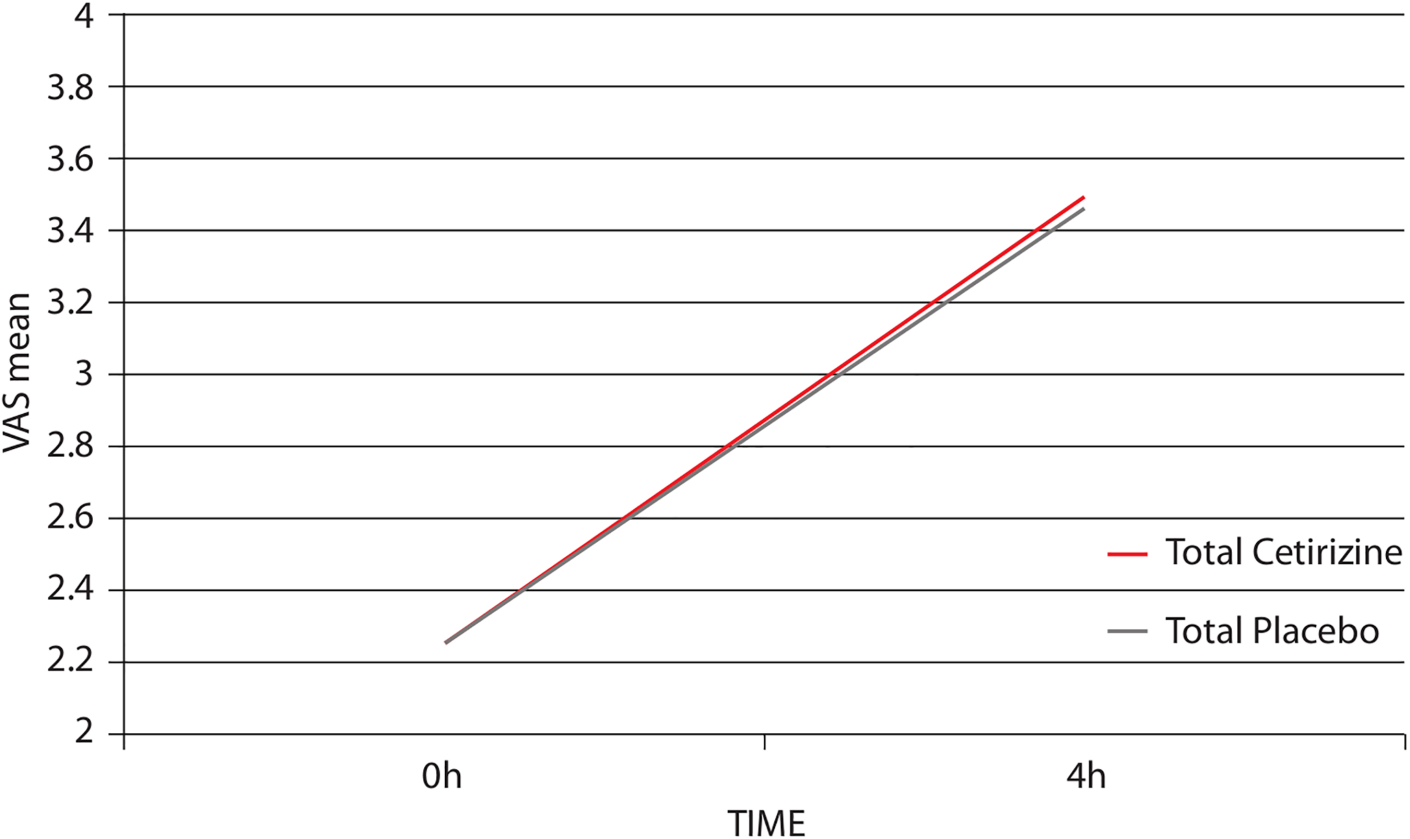
Change of FLS severity (VAS) from before (0 h) to after interferon-ß injection (4 h) (primary endpoint). Visual analog (VAS) scores were collected at 4 weeks in each treatment sequence. Data shown are total mean VAS scores. ANCOVA analysis was performed on the ITT population (N = 39).

The efficacy analysis was performed on the ITT population (N = 42), however only 39 patients were included as data were missing for 3 patients. The differences between cetirizine and placebo in the primary endpoint (the mean change of FLS severity after 4 weeks of therapy within each sequence) were not statistically significant (Fig 2). Total mean VAS scores at 4 hours from IFNβ injections were 3.57 and 3.42 for cetirizine and placebo, respectively (difference –0.15; 95% CI: from –0.74 to 0.44; p = 0.6029) (Table 2). Overlapping results were obtained when a Last Observation Carried Forward (LOCF)-, or a Next Observation Carried Backward (NOCB)-analysis were performed.

**Table 2:** Mean VAS scores of FLS collected 4 hours after interferon-β (IFNß) injection on the fourth week of each treatment sequence.

Sequence	Cetirizine	Placebo	Difference	p-value
**Placebo-Cetirizine**	3.15 (2.95)	3.45 (3.09)	0.30 (2.60)	-
**Cetirizine-Placebo**	3.97 (2.58)	3.37 (2.35)	–0.60 (2.53)	-
**Total**	3.57 (2.76)	3.41 (2.71)	–0.15 (1.81) 95% CI: –0.74, 0.44	0.6029

The analysis of the primary endpoint was performed on the ITT population (3 patients were excluded due to missing data, thus N = 39). Data are shown as mean values (± standard deviation)

CI = Confidence interval

The analysis of the secondary endpoints (mean changes in FLS severity; proportions of responders; incidence of FLS) also failed to detect statistically significant differences between cetirizine and placebo. Mean changes in FLS severity (as assessed by VAS scores and FLS-S) were similar with cetirizine and placebo throughout the observation period (Table 3); no statistically significant differences were seen within treatments either.

**Table 3:** Comparison of FLS severity (VAS and FLS-S) with placebo and cetirizine 4 hours and 12 hours after interferon-β (IFNß) injection (secondary endpoint).

	VAS		FLS-S	
	mean difference (±SD) placebo-cetirizine	p-value	mean difference (±SD) placebo-cetirizine	p-value
**1**^**st**^ **week 4h**	0.1 (0.4)	0.7258	0.0 (0.5)	0.9559
**1**^**st**^ **week 12h**	0.2 (0.4)	0.6493	–0.2 (0.5)	0.6984
**2**^**nd**^ **week 4h**	0.6 (0.4)	0.1191	1.0 (0.5)	0.0313
**2**^**nd**^ **week 12h**	0.7 (0.4)	0.0743	0.3 (0.5)	0.5799
**3**^**rd**^ **week 4h**	–0.3 (0.4)	0.4685	–0.4 (0.5)	0.4065
**3**^**rd**^ **week 12h**	–0.1 (0.4)	0.8186	0.0 (0.5)	1.0000
**4**^**th**^ **week 4h**	0.0 (0.4)	0.9274	–0.3 (0.5)	0.5426
**4**^**th**^ **week 12h**	0.2 (0.4)	0.5001	–0.1 (0.5)	0.8248

FLS-S = flu-like syndrome symptom score

SD = standard deviation

VAS = visual analog scale

The study was however not powered to detect statistically significant differences with regard to these efficacy measures. The proportions of responders were numerically greater with cetirizine versus (vs.) placebo at 4 hours (35% vs. 25%) and 12 hours (50% vs. 35%) after injection, however the differences between groups were not statistically significant. As for the incidence of FLS, 80% of patients treated with cetirizine and 82.5% of patients treated with placebo experienced a flu-like event (i.e., increase ≥2 FLS-S compared with the value before injection) at 4 hours (difference between treatment not statistically significant). At 12 hours, such proportions were 70% for both treatments.

Included in the safety population were all patients who received at least one dose of cetirizine (N = 45). Overall, 16 patients (35.6%) experienced adverse events. Of the 20 adverse events registered, all of mild to moderate intensity, none was classified as treatment-related. No serious adverse events were reported and no patient interrupted treatment due to adverse events.

As for adverse events commonly associated with the administration of cetirizine, patients did not experience excessive daytime sleepiness, as assessed by the ESS. After adjustment for sleepiness at baseline, ESS scores were not significantly different between placebo and cetirizine [mean (±SD) total ESS score of placebo vs. cetirizine, 4.2 (±3.9) vs. 5.0 (±4.7), difference –0.8 (±3.0), 95% confidence interval from –1.8 to 0.2, p = 0.098].

## Discussion

This was to our knowledge the first study addressing the efficacy of a second-generation H1 histamine receptor antagonist on IFNβ-induced FLS in patients with RRMS. The addition of cetirizine to the standard of care for FLS failed to provide a significant benefit compared with placebo. The findings from this pilot study, therefore, do not seem to support our hypothesis that cetirizine, via its multiple modulatory effects on allergic and inflammatory responses that include the down-regulation of IL-6, might alleviate IFNβ-induced FLS in RRMS patients not adequately controlled by paracetamol or NSAIDs.

The adequate treatment of IFNβ-induced FLS remains an unmet need in the management of patients with RRMS. FLS, though manageable in many cases, continues to be a common cause of poor adherence to IFNβ therapy and early treatment discontinuation, an issue perceived also by patients [17–19]. In fact, according to a recent analysis of patient preferences for features of injectable disease modifying therapies for multiple sclerosis, treatment efficacy seems as important as reduction in injection frequency or reduction in some adverse events for patients using injectable medications [20]. The relationship between adherence to IFNβ therapy and the rates of multiple sclerosis relapse is well established. For example, a recent study comparing relapse rates and healthcare resource utilization in adherent and non-adherent patients found that the IFNβ-adherent group tended to have a lower risk of relapse over a 3-year period than the non-adherent group [4]. Adherent patients also had a lower risk of visits to the emergency department and hospital admission over the same period [4].

The severity of FLS varies considerably between and within patients. This characteristic of FLS was observed also in our patient population. This great variability might explain, at least in part, the absence of a significant effect of cetirizine compared with placebo on FLS. Also, the reported severity of FLS was mild to moderate on average, which might have precluded the detection of an effect caused by cetirizine. Studies in selected patient populations with a more homogeneous FLS status, and with moderate to severe symptom severity, might be more adequate for investigating the efficacy of a treatment in relieving flu-like symptoms.

A further limitation of our study was the heterogeneity of IFNβ formulations used (intramuscular IFNβ-1a, requiring 1 injection weekly; subcutaneous IFNβ-1a, 3 injections weekly; subcutaneous IFNβ-1b, 1 injection on alternate days), although it must be pointed out that about 70% of our patients used the same IFNβ formulation, namely subcutaneous IFNβ-1a 44 μg. Different IFNβ administration modes resulted in different cetirizine dosages, according to the study protocol. Differences in the frequency of FLS with different IFNβ administration modes cannot be excluded either, as the occurrence of FLS appears to correlate with the frequency of IFNβ injections. With regard to the serum IFNβ levels achieved with different formulations, a recent review of the pharmacokinetic data of the three formulations used in the present study showed that the occurrence of flu-like symptoms after IFNβ injection clearly correlates with an increase of IFNβ serum concentrations [21]. According to this review, however, the pharmacokinetic characteristics of the different types of IFNβ and routes of administration are similar.

The time of IFNβ injection appears to affect the severity of flu-like symptoms. A study comparing the time course of plasma hormone and cytokine levels and the severity of side effects in RRMS patients injecting IFNβ in the morning or in the evening found that IFNβ administration in the evening resulted in a more rapid increase in IL-6 plasma levels and temperature and was associated with more severe symptoms compared with morning administration [22]. In a more recent study, the change of the IFNβ injection time from evening to morning improved flu-like symptoms and sleep in a substantial proportion of the study cohort consisting of RRMS patients with persistent IFNβ-related FLS [23]. Our patients were all taking IFNβ in the evening, according to current recommendations, with some patients injecting it in the early evening and others in the late evening. Whether this difference might have had an impact on FLS is currently unclear.

In the present study, cetirizine 10 mg was administered 1 hour before each injection of IFNβ. The pharmacodynamic effect of cetirizine on IL-6 has not been studied in detail and it is currently unknown whether this dosage was sufficient to elicit an effect of cetirizine on IL-6 production, especially in patients on high-dose IFNβ. Cetirizine has a rapid onset of action (15–180 minutes), with its effects peaking at 4–8 hours and persisting for at least 24 hours [13,24]. Thanks to these characteristics, cetirizine can be used on-demand for the treatment of clinical symptoms of allergic disorders, but it has been suggested that continuous administration (10 mg, once daily) may be necessary to achieve better efficacy by reducing also the underlying allergic inflammation [25]. A continuous administration of cetirizine may also be required for the control of FLS, at least during the first months of IFNβ therapy.

In a recent attempt to identify genetic factors responsible for the occurrence of FLS, we analyzed whether a single nucleotide polymorphism in the promoter region of the IL-6 gene, known to affect IL-6 levels, would correlate with the incidence of FLS [26]. Patients carrying at least one copy of the -174 G>C polymorphism expressed lower levels of IL-6 and were also found to be less prone to develop FLS. Furthermore, in these patients, FLS was less severe. Thus, the presence of at least one C allele at position -174 of the IL-6 gene determines a lower likelihood of developing IFNß-related events. It is unknown how many patients in the present study population presented this polymorphism. Additionally, as this was a pilot clinical study we did not plan to investigate the pharmacodynamics of cetirizine in counterbalancing the release of IL-6 induced by IFNß, by measuring IL-6 before and after treatment. This would have helped in the interpretation of results and represents a limitation of the study to be implemented in future evaluations.

Future studies in patients selected based on their predisposition to develop IFNß-related FLS might contribute to increase our understanding of the mechanisms underlying this common adverse event and may help design effective prophylactic and therapeutic strategies.

## Conclusions

The addition of a second-generation histamine H1 receptor antagonist to the standard of care for IFNβ-induced FLS in patients with RRMS does not seem to improve symptoms significantly compared with placebo. FLS continues to be inadequately treated in many RRMS patients. Further investigations are needed to elucidate the underlying mechanisms of IFNβ-induced FLS and develop adequate strategies for prevention and treatment.

## Supporting information

S1 Checklist CONSORT 2010 checklist. (PDF)

S1 File Final study protocol. (PDF)

S1 Data (XLSX)
