# Supplementary material for: Management of flu-like syndrome with cetirizine in patients with relapsing-remitting multiple sclerosis during therapy with interferon beta: Results of a randomized, cross-over, placebo-controlled pilot study
Source: PLoS One. 2017 Jul 7;12(7):e0165415. doi: 10.1371/journal.pone.0165415 (PMC5501388; doi:10.1371/journal.pone.0165415)
Supplement: S1 File — (PDF) [file pone.0165415.s002.pdf]

FLU-LIGHT Study

Flu Like Inhibition Giving anti-Histaminic Therapy

A monocentric, randomised, placebo-controlled, double-blind crossover study to evaluate the efficacy of cetirizine on Flu-Like syndrome (FLS) in multiple sclerosis patients treated with interferon-β

Protocol BIIT 0212

Final version No. 1 of 12/04/2013

EudraCT Code: 2013-001055-12

Study phase: pilot study, phase III b

Study Drug: Cetirizine

This document, along with all the information and data contained therein, shall be considered and treated as strictly confidential. Any information contained therein may not be disclosed to anyone without prior written consent of Biogen Idec Italia S.r.l.

TABLE OF CONTENTS

1 OUTLINE 4
2 CENTRE, STAFF AND SIGNATURES 8
2.1 Roles 8
2.2 Immediate reporting of Serious Adverse Events (SAEs)..9
2.3 Authorisations and signatures 10
3 INTRODUCTION AND RATIONALE 12
4 STUDY OBJECTIVES 13
5 STUDY PLAN 13
5.1 Study design and operational plan outline 13
5.2 Rationale behind the study design..14
5.3 Study duration 14
6 STUDY POPULATION 14
6.1 Inclusion criteria 14
6.2 Exclusion criteria..15
6.3 Withdrawal of subjects from the study 15
7 STUDY PROCEDURES..16
7.1 Flow chart..16
7.2 Observation period 16
7.3 Evaluations 18
8 STUDY TREATMENTS..19
8.1 Treatments to be administered 19
8.2 Identity of the study products 19
8.2.1 Packaging and labelling..19
8.3 Storage and delivery of the study drug 20
8.4 Randomisation procedure 21
8.5 Blinding 21
8.6 Adherence to the treatment..22
8.7 Previous treatments and concomitant therapies..22
8.9 Side effects of the study drug..23
9 Pharmacovigilance..23
9.1 Definitions 23
9.1.1 Adverse event 23
9.1.2 Serious adverse event..23
9.2 Recording 24
9.3 Correlation judgement 24
9.4 Adverse Drug Reaction (ADR) 24
9.5 Unexpected Adverse Drug Reaction..24
9.6 Notification of serious adverse events 24
10 DATA MANAGEMENT AND STATISTICAL ANALYSIS 25
10.1 Data management 25
10.2 Sample size..25
10.3 Statistical analysis..26
10.3.1 Descriptive analysis 26

10.3.2 Inferential statistics ... 26
10.3.3 Uniformity of the sequences to the baseline ... 26
10.3.4 Efficacy ... 26
10.3.5 Compliance ... 27
10.3.6 Adverse events ... 27
10.3.7 Data recording ... 27
10.3.8 Criteria for evaluation of data ... 27
10.3.9 Withdrawal of patients from the study ... 28
11 ACCESS TO ORIGINAL DATA/DOCUMENTS ... 28
12 MONITORING ... 28
13 AMENDMENTS ... 28
14 ETHICS/REGULATORY REQUESTS ... 28
14.1 Study authorisation ... 29
15 INFORMED CONSENT FORM AND INFORMATION LEAFLET ... 29
16 STUDY DISCONTINUATION ... 30
17 FINAL REPORT ... 30
18 DOCUMENT STORAGE ... 30
19 INSURANCE COVERAGE ... 30
20 FINANCIAL TERMS AND CONDITIONS ... 31
21 USE OF INFORMATION AND PUBLICATION OF DATA ... 31
22 REFERENCES ... 32

1 OUTLINE

**Study title**	FLU-LIGHT Study -**Flu L**ike **I**nhibition **G**iving anti-**H**istaminic **T**herapy.<br>A monocentric, randomised, placebo-controlled, double-blind crossover study to evaluate the efficacy of cetirizine on Flu-Like syndrome (FLS) in multiple sclerosis patients treated with interferon-β
**Study code and Phase**	BIIT 0212, pilot study phase IIIb.<br>This is an interventional study that will be conducted in accordance with AIFA (Agenzia Italiana per il Farmaco [Italian Medicines Agency (IMA)]) and European clinical trials directives. The study is registered with EudraCT number 2013-001055-12.
**Study duration**	The study will be conducted for at least 20 weeks (12 weeks for the enrolment period and 8 weeks for the treatment); each patient shall participate in the study for an overall period of 8 weeks of treatment with the study drug (4 with the active drug and 4 with a placebo).
**Objectives**	Main objective:<br>• Evaluate the efficacy of cetirizine 10 mg in reducing flu-like syndrome symptoms in patients with RRMS treated with IFNβ.
**Study Drug**	All patients already being treated with IFNs will be randomised into two sequences with cetirizine 10 mg and/or placebo, in a crossover study.<br>40 patients in treatment with IFN randomised into two sequences:<br>• Sequence 1 (20 patients):<br>• first period (4 weeks) placebo<br>• second period (4 weeks) cetirizine.<br>• Sequence 2 (20 patients):<br>• first period (4 weeks) cetirizine<br>• second period (4 weeks) placebo
**Treatment period**	8 weeks. After this period patients will continue their existing IFNβ treatment.<br>For each IFNβ injection<br>• Baseline<br>FLS-VAS, Record symptoms and body temperature<br>Administer the study drug<br>**After one hour:**<br>Administer IFNβ<br>• After 4 hours (Time A)<br>FLS-VAS, Record symptoms and body temperature<br>• After 12/15 hours (Time B)<br>FLS-VAS, Record symptoms and body temperature

<table>
<tr><td>Study design</td><td>A randomised, monocentric, placebo-controlled, double-blind, crossover phase IIIb pilot study.</td></tr>
<tr><td>Inclusion/
Exclusion criteria</td><td><u>Inclusion criteria</u>
• Patients of both genders.
• Patients between the ages of 18 and 55 years old.
• Patients with Relapsing-Remitting Multiple Sclerosis (RRMS).
• IFNβ 1a treatment (IM or SC) or 1b for at least 3 months.
• Pregnancy test with negative result carried out no more than 30 days from the baseline visit (if applicable)
• FLS-S ≥ 2 despite standard therapy (SOT) for FLS.
• Ability to provide written informed consent for participating in the study.
• Patients must not present with clinically significant conditions or circumstances, with the exception of MS, which in the opinion of the Investigator may interfere with study evaluations or participation in the study.
• Use of effective contraception or being in menopause for at least 6 months (where applicable)
<u>Exclusion criteria</u>
• Potentially fertile patients (male or female) who are not using contraception.
• Women who are pregnant or breastfeeding.
• Known intolerance or contraindications for cetirizine use.
• Known hereditary condition of intolerance to galactose, Lapp lactase deficiency, glucose-galactose malabsorption
• Simultaneous participation in other clinical studies.</td></tr>
<tr><td>Sample size</td><td>The severity of the FLS expressed as the patient's judgement on a visual analogue scale (VAS) ranging between 0 and 10 is the parameter used to calculate the sample.

The clinical hypothesis is that four hours after administering IFN, the average difference (d) of the VAS score between the two treatments is equal to or greater than 0.50 where d is expressed in standardized units.

Selecting $\alpha = 0.05$ and $\beta = 0.20$ (power =80%), it is necessary to enrol 34 patients, who will be sequentially treated in accordance with a crossover design, as shown in the randomisation list. Taking into consideration the possibility of a 15-20% drop-out rate, the sample to be randomised for the two sequences will be made up of 40 patients.</td></tr>
<tr><td>Statistical analysis</td><td>The observations will be made before the IFN injection, 4 hours after and 12/15 hours after the injection.
• Descriptive analysis
All the variables recorded in the Case Report Form will be reported in descriptive tables. In particular, continuous variables will be reported as a mean and SD, while nominal or discrete variables will be summarized in contingency tables.
• Uniformity of the sequences to the baseline.</td></tr>
</table>

	• Evaluation of the efficacy variables.<br><br>The severity of the FLS shall be evaluated using the FLS- VAS score and the symptom score (FLS-S), taken from the composite score of the following symptoms: muscle pain, chills and weakness as well as body temperature score, as described in the protocol. The FLS-VAS and FLS-S score variables will be analysed using analysis of variance (ANOVA) with repeated measurements. In accordance with the crossover design.<br><br>• Tolerability.<br><br>Adverse event frequency will be analysed descriptively. In particular, we will analyse the information about treatment compliance and FLS symptoms recorded in the patient diary.
**Sponsor**	Biogen Idec Italia S.r.l. – Centro Leoni Edificio A<br><br>Via Spadolini, 5<br><br>20141 Milan

LIST OF ABBREVIATIONS

AIFA	(Agenzia Italiana per il Farmaco [Italian Medicines Agency])
EC	Ethics Committee
PIC	Personal Identity Code
RandCod	Randomisation Code
CRF	Case Report Form
CRO	IBIS Informatica Srl + Clinical Trial Consulting (CTC) ss + Corden Pharma SpA
UCTS	Unique code for trial subject
DMT	Disease Modifying Therapy
AE	Adverse Event
EMA	European Medicines Agency
NSAIDS	Nonsteroidal Anti-Inflammatory Drugs
FLS	Flu-like syndrome
FLS-S	Flu-Like syndrome score
HQoL	Health Quality of Life
IFN	Interferon
IL-6	Interleukin 6
RN	Randomisation number
SAE	Serious Adverse Event
RRMS	Relapsing-Remitting Multiple Sclerosis
SOT	Standard of Therapy
TMF	Trial Master File
TNF	Tumour Necrosis Factor
FLS-VAS	Visual Analogue Scale for Flu-Like syndrome

2 CENTRE, STAFF AND SIGNATURES

One single centre will participate in this study in Italy.

2.1 Roles

Sponsor	Biogen Idec Italia S.r.l. – Centro Leoni Edificio A<br>Via Spadolini, 5 – 20141 Milan<br>Tel.: +39 02 584991 - Fax: +39 02 58383477<br>Study Director:<br>Dr Guido Sabatella<br>e-mail: guido.sabatella@biogenidec.com
Centre	Tor Vergata University Hospital –<br>Centro Sclerosi Multipla [Multiple Sclerosis Centre]<br>Viale Oxford, 81 - ROMA - 00133<br>Tel.: +39 06 2090 3111 - Fax: +39 06 7259 6006<br>Study Director<br>Prof. Diego Centonze<br>e-mail: centonze@uniroma2.it
CRO	IBIS Informatica S.r.l.<br>Via Carlo D'Adda, 8.- 20143 Milan<br>Director Dr Duilio Ferrari (Scientific Director)<br>Tel, +39 02 8330 151<br>e-mail: duilio.ferrari@ibisinformatica.com

2.2 Immediate reporting of Serious Adverse Events (SAEs)

All Adverse Events (AE) that occur during the study must be reported in the proper section of the e-CRF. All Serious Adverse Events (SAEs) and any pregnancies that occur during the study must be reported within one business day (24 hours) to Biogen Idec Italia. The Investigator shall provide the basic information (Study number, subject identification code, date of birth, gender, IFN treatment and randomisation code, nature of SAE and relation with the study drug)

SAE reports should be sent to:

Roberta Amodeo

Senior Associate I, Drug Safety

Biogen Idec Italia S.r.l. – Centro Leoni Edificio A

Via Spadolini 5 – 20141 Milano

Tel. +39 02 58499043 – Mobile. +39 3425772172

Fax: +39 02 58499134

e-mail: farmacovigilanza@biogenidec.com

2.3 Authorisations and signatures

FLU-LIGHT Study

AGREEMENT

By signing this document, the Principal Investigator declares he/she has read and understood the Protocol, which contains all the ethical, legal and scientific information needed to conduct the study. The Principal Investigator will personally conduct the study as set out in the Protocol and in accordance with the Declaration of Helsinki (as amended), the International Conference on Harmonisation (ICH) Guideline for Good Clinical Practice (GCP) (Committee for Proprietary Medicinal Products [CPMP]/ICH/135/95) adopted in Italy within the framework of Ministerial Decree of 15/07/1997, EU Directive 2001/20/EC adopted in Italy within the framework of Legislative Decree 211 of 24/06/2003, EU Directive 2005/28/EC adopted in Italy within the framework of Legislative Decree 200 of 06/11/2007, the Good Manufacturing Practice (GMP) Directive 2003/94/EC, and all applicable legal requirements, including any subsequent amendments.

The Principal Investigator shall provide copies of the protocol to all investigators, nurses and other professional staff appointed to participate in the Study. The Principal Investigator is aware of the fact that this Protocol must be approved by his/her Ethical Committee, to which it will be submitted for evaluation by the Sponsor/CRO.

By signing this agreement, the Investigator agrees that the clinical data entered into the electronic Case Report Forms (e-CRFs) by him/her or members of his/her appointed staff will be used by the Sponsor for the purpose of the research. In addition, he/she authorises inspections by staff appointed by the Sponsor/CRO as well as inspections by the Regulatory Authorities, and full access to all the medical records at the research centre for the screened or randomised study subjects.

The Investigator agrees to provide all subjects with a written informed consent form, as required by ICH-GCP Guidelines and current legislation on clinical trials.

By signing this Agreement, the Investigator agrees not to publish or disclose any information contained therein without prior written consent of Biogen Idec Italia S.r.l.

However, this document may be disclosed to the competent Ethics Committees or authorised representatives of the Investigator or the health authorities, on the condition that they also be required to respect the confidentiality of this document. Publication (oral or written) of partial or final data is subject to prior agreement between the Investigator and Biogen Idec Italia S.r.l.

**Biogen Idec Italia s.r.l.**<br>GUIDO SABATELLA<br>Signature<br>____<br>Biogen Idec Italia Srl	Date<br>___/___/_____
**Principal Investigator**<br>DIEGO CENTONZE<br>Signature<br>____<br>Trial Centre no. 01<br>Tor Vergata University Hospital – Multiple Sclerosis Centre	Date<br>___/___/_____
**CRO Study Manager**<br>DUILIO FERRARI<br>Signature<br>____<br>IBIS Informatica & Idee srl	Date<br>___/___/_____

3 INTRODUCTION AND RATIONALE

Multiple sclerosis (MS) is an autoimmune disease of the central nervous system characterised by relapses and progressive disability. The treatment of Relapsing-Remitting Multiple Sclerosis (RRMS) is based on therapies capable of modifying its natural course (disease-modifying therapies- DMTs); first-line therapies include interferon beta-1a (IFNβ-1a), IFNβ-1b and glatiramer acetate (GA) (Galetta et al., 2002).

The most frequent side effect of IFNβ is flu-like syndrome (FLS) which includes myalgia, muscle weakness, chills and fever (Galetta and Markowitz 2005). Symptoms generally begin 3-6 hours after the injection and resolve within 24 hours. FLS, generally present in the first few months of therapy (Mohr et al., 1998), is the most frequent reason for discontinuing the treatment (Tremlett and Oger 2003).

A strategy to reduce the frequency and intensity of FLS is dose titration when IFNβ treatment is started (Brandes et al., 2007). Low doses of steroids, paracetamol and non-steroidal anti-inflammatory drugs (NSAIDs) such as ibuprofen have proven to be effective in reducing the frequency and symptoms of FLS (Rio et al., 1998, Rice et al., 1999, Reess et al., 2002, Rio et al., 2004).

Despite these strategies, FLS persists at unacceptable levels in some patients and it becomes necessary to discontinue the therapy (Tremlett and Oger 2003). Management of FLS is therefore considered to be an unmet clinical need.

The pathogenesis of the symptoms needs to be understood in order to try to further reduce the impact of FLS thereby allowing the patient to continue the therapy and optimize its effectiveness. FLS appears to be linked to increases in endogenous pyrogens such as Interleukin 6 (IL-6) and Tumour Necrosis Factor-alpha (TNF-α) (Roth et al., 1997). An increase in IL-6 levels has been observed in patients who develop FLS during the first weeks of therapy; the increase is lower in patients treated with low doses of oral steroids (Martinez-Caceres et al., 1998, Rio et al., 2004). Moreover, a stimulation study with IFNβ-1b has shown that the leukocytes of patients who develop fever as a result of therapy generally produce more interleukin-6 in vitro following a stimulation study with IFNβ. (Montalban et al., 2000).

Cetirizine, a third-generation antihistamine, is a hydroxyzine metabolite and a potent selective antagonist of peripheral H1 receptors, used in the treatment of allergies, hay fever, angioedema and urticaria (EMA 2008). Compared to first and second generation antihistamines, cetirizine has less sedative effects. In vitro studies have shown that second and third generation H1 antagonists inhibit the release or production of mediators of inflammation such as IL-6; they also reduce the chemotaxis and adhesion of eosinophils (Marone et al., 1999, Marone et al., 2001, Triggiani et al., 2001).

Considering the in vitro effects on IL-6, cetirizine treatment could reduce FLS associated with IFNβ therapy.

4 STUDY OBJECTIVES

To evaluate the effectiveness of the administration of cetirizine 10 mg in improving FLS symptoms.

The primary objective of the study is the mean variation in subjective severity of the FLS, evaluated according to the patient's judgment expressed on a visual analogue scale (FLS-VAS).

The secondary objectives are: the mean variation in severity of the FLS (evaluated based on the score of FLS symptoms (Flu-Like syndrome score (FLS-S))) between the findings before the injection, 4-6 hours after the injection and 12-15 hours after the injection of IFNβ during the study period; the proportion of patients with a mean reduction of ≥2 in FLS-S; and the incidence of FLS (defined as an increase of 2 or more FLS-S points compared to the value before the injection) 4-6 hours after the injection and 12-15 hours after the injection during the study period.

The safety and tolerability of the drug will be monitored for the entire duration of the study; in particular the most common adverse reaction to cetirizine (sleepiness), will be evaluated using the Epworth Sleepiness Scale (ESS) (Johns et al., 1991), validated in Italian (Vignatelli et al., 2003), at the baseline visit (V1), at 4 (V2) and 8 (V3) weeks (or at the final study visit - Vx).

5 STUDY PLAN

5.1 Study design and operational plan outline

A randomised, monocentric, placebo-controlled, double-blind, crossover phase IIIb pilot study.

The patients will be randomised into a period of standard therapy plus cetirizine 10 mg lasting 4 weeks followed by 4 weeks of standard therapy plus placebo, or else in the opposite sequence. If the patient is already taking drugs for the treatment of FLS, for example paracetamol or ibuprofen, they must continue to maintain the dose of that therapy as stable as possible during the study period; this is defined by the protocol as the reference standard of therapy (SOT), at the discretion of the Investigator and in accordance with standard clinical practice.

The subjective severity of the FLS shall be evaluated by the patient expressed on a visual analogue scale (FLS-VAS). The FLS-VAS scale is 10 cm in length where 0 = No discomfort due to FLS and 10 = Maximum conceivable discomfort due to FLS.

Flu-Like Syndrome symptoms (muscle pain, chills and weakness) and body temperature will be evaluated using the FLS-S score.

To calculate FLS-S, patients must assign a score to the presence and intensity of muscle pain, chills and weakness, each separately, according to the following scale: 0 = absent; 1 = minor, has not interfered with daily activities; 2 = moderate, has interfered with normal activities; 3 = severe, requiring bedrest. In addition, the patient's body temperature will be recorded to ascertain whether fever is present using the following scale: 0: <37.3°C; 1:≥37.3°C and <37.8°C; 2: ≥37.8°C and <38.4°C; 3: ≥38.4°C. The scores for individual symptoms (muscle pain, chills, weakness and body temperature) will be added together to calculate the FLS-S, which will therefore vary between 0 and 12. A change in the total score ≥ 2 points above the pre-injection score has been predefined as positive for FLS.

All scores (FLS-VAS and FLS-S) will be recorded at three distinct times for each IFNβ injection: baseline (when taking the study drug, 1 hour before the injection); Time A (4 hours after the

injection) and Time B (12-15 hours after the injection). All the scores and any analgesic doses taken will be noted in the patient diary.

The patients will record the data for each injection for 8 weeks.

5.2 Rationale behind the study design

Flu-like symptoms are largely subjective, and symptoms such as muscle pain, chills and a state of exhaustion may be associated with significant subjective variability which, combined with a different response to therapy, can create a confounding effect that is difficult to distinguish from the effect of the drug and the placebo.

Under these conditions, adopting an experimental parallel-arm design would entail an excessively large sample of subjects in order to have sufficient statistical power.

The advantage of a crossover approach lies in a smaller sample size, as every subject is exposed to a period of placebo treatment as well as a period of treatment with the study drug in a randomised sequence. Therefore each patient contributes to the sample size for more than one treatment, and the variability of intra-subject measurements is smaller than that between different subjects, revealing more clearly the difference between the drug and the placebo.

A crossover design is a type of randomised clinical trial in which N experimental units are divided into 2 groups: those randomly assigned to the first group receive the placebo and then the antihistamine; those randomised to the second group receive the antihistamine first and then the placebo. Each time interval in which one of the two treatments is administered is called a period.

In crossover designs in which two active drugs are compared, a suitable wash out time needs to be introduced between the end of one treatment and the start of the next to avoid carrying over the effect of one treatment into the next period.

On the other hand, when one of the two treatments is a placebo, this interval is not introduced as the wash out would simply lengthen the placebo treatment time. In this case the passage from one treatment to the other can occur without any interruption.

5.3 Study duration

Overall, the study will last for 20 months. Patient recruitment is expected to take 12 weeks starting from the date the centre opens once the authorisation process is complete, and 8 weeks of treatment.

6 STUDY POPULATION

The study will enrol 40 patients with Relapsing-Remitting Multiple Sclerosis (RRMS) being treated with IFNβ 1a (IM or SC) or 1b (SC) for at least 3 months who present with FLS despite being treated with paracetamol or NSAIDs, defined by the protocol as standard therapy (standard of therapy, SOT) for FLS.

6.1 Inclusion criteria

To be eligible, candidates must meet all of the following inclusion criteria at the start of the study:

- Patients of both genders.
- Patients between the ages of 18 and 55 years old.
- Patients with Relapsing-Remitting Multiple Sclerosis (RRMS).
- IFNβ 1a treatment (IM or SC) or 1b for at least 3 months.
- Pregnancy test with negative result carried out no more than 30 days from the baseline visit (if applicable).
- FLS-S ≥ 2 despite standard therapy (SOT) for FLS.
- Subjects who do not present with clinically significant conditions or circumstances, with the exception of MS, which in the opinion of the Investigator may interfere with study evaluations or participation in the study.
- Ability to provide written consent for participating in the study.
- Use of effective contraception or experiencing menopause for at least 6 months (if applicable).

6.2 Exclusion criteria

- Potentially fertile subjects (whether male or female) who are not using contraception.
- Women who are pregnant or breastfeeding.
- Known intolerance or contraindications for cetirizine use.
- Known hereditary condition of intolerance to galactose, Lapp lactase deficiency, glucose-galactose malabsorption.
- Simultaneous participation in other clinical studies.

6.3 Withdrawal of subjects from the study

Each patient shall have the right to freely withdraw from the study at any time, with no obligation to give a reason, and without prejudice to future care from the medical personnel and/or Hospital.

The Investigator or the Sponsor also have the right to exclude subjects from the study in the event of a change in eligibility criteria or for other reasons.

In the event subjects withdraw from the Study, for any reason, the Investigator must fill in the relevant section in the e-CRF, indicating the reason for the patient's premature withdrawal from the study.

Withdrawal may occur due to:

- Discontinuation of therapy.
- Patient failure to return to the authorised Study centre.
- Patient withdrawal of consent for participating in the Study.

7 STUDY PROCEDURES

7.1 Flow chart

Procedures	Visit 1 (V1) (T=0)	Visit 2 (V2) (T=4 weeks)	Visit 3 (V3) (T=8 weeks)	Drop-out visit (Vx) (T=x)
**Informed Consent**	**X**			
**Inclusion/Exclusion Criteria**	**X**			
**Randomisation**	**X**			
**General medical history**	**X**			
**MS medical history**	**X**			
**Neurological examination and EDSS calculation**	**X**	**X**	**X**	**X**
**Verification of concomitant therapies**	**X**	**X**	**X**	**X**
**Verification of current IFNβ treatment**	**X**	**X**	**X**	**X**
**Epworth sleepiness scale (ESS)**	**X**	**X**	**X**	**X**
**Recording of adverse effects (including relapses)**		**X**	**X**	**X**
**Diary given to the patient**	**X**	**X**		
**Diary collected from the patient**		**X**	**X**	**X**
**Drug provided**	**X**	**X**		
**The unused drug is collected and counted**		**X**	**X**	**X**

7.2 Observation period

Patients will be followed for 8 weeks after their enrolment in the Study.

Each patient will be required to attend 3 visits in the following time frame:

- at the enrolment visit (Visit 1 – V1);
- 4 weeks after the enrolment visit (Visit 2 – V2);
- 8 weeks after the enrolment visit (Visit 3 – V3)

The diary for the first 4 weeks of treatment (1st - 4th week, first month), including FLS-VAS and FLS-S, will be given to the patient at Visit 1, with verification of the patient diary completeness and collection taking place at Visit 2. For the next 4 weeks of treatment (5th - 8th week, second

month), the diary will be given to the patient at Visit 2, checked for completion and collected at Visit 3. Patients will be requested to fill out the diary after each injection of IFNβ for the duration of the study (8 weeks).

At visits V1, V2 and V3 the patient will fill in the Epworth Sleepiness scale (ESS) questionnaire with help from the Investigator.

In the case of premature withdrawal from the study, the patient will be asked at visit Vx to fill in the questionnaire and the Investigator will collect the diary provided at visit V1 or V2, and will verify that it was correctly filled in.

To summarise, the following procedures will be carried out:

ENROLMENT VISIT - V1 (T0)

- Signing of Informed Consent
- Verification of inclusion/exclusion criteria
- Randomisation
- Collection of general clinical history (General medical history)
- Collection of MS clinical history (MS History)
- Neurological examination and EDSS calculation
- Verification of concomitant therapies
- Verification of current IFNβ treatment
- Epworth sleepiness scale
- Diary given to patients
- Drug provided

VISIT 2 - V2 (Week 4)

- Neurological examination and EDSS calculation
- Verification of concomitant therapies
- Confirmation of current IFNβ treatment
- Epworth sleepiness scale
- Recording of adverse effects (including relapses)
- Diary given to patients
- Collection and verification of the patient diary completeness
- Drug provided
- The unused drug is collected and counted

VISIT 3 - V3 (Week 8 or end of study)

- Neurological examination and EDSS calculation
- Verification of concomitant therapies
- Confirmation of current IFNβ treatment

- Epworth sleepiness scale
- Recording of adverse effects (including relapses)
- Collection and verification of the patient diary completeness
- The unused drug is collected and counted

DROP-OUT VISIT - VX (TX)

In the case of premature withdrawal of the patient from the study, it is advisable, if possible, to carry out all recordings and examinations planned for the final visit (V3) during the Vx visit.

- Neurological examination and EDSS calculation
- Verification of concomitant therapies
- Confirmation of current IFNβ treatment
- Epworth sleepiness scale
- Recording of adverse effects (including relapses)
- Collection and verification of the patient diary completeness
- The unused drug is collected and counted

7.3 Evaluations

Completion of the questionnaire (Epworth Sleepiness Scale – ESS)

The Epworth Sleepiness Scale (ESS) questionnaire is validated in Italian (Vignatelli et al., 2003) and contains the patient's last name, first name and randomisation code. These documents will not be collected by the Monitor but they will be an integral part of the documentation of the centre where the study is conducted. Questionnaire data, filled in by patients on paper at visit V1, V2 and V3, will be recorded in the electronic Case Report Form (e-CRF) by the Investigator.

Filling in diaries

The diary for the first 4 weeks of treatment (1st - 4th week, first month), will be given to the patient at Visit 1, with verification of the patient diary completeness and collection taking place at Visit 2. For the next 4 weeks of treatment (5th - 8th week, second month), the diary will be given to the patient at Visit 2, checked for completion and collected at Visit 3. Patients will be requested to fill out the diary after each injection of IFNβ for the duration of the study (8 weeks).

In the case of premature withdrawal from the study by a patient who is able to attend a final visit at the centre, the Investigator will collect the diary handed out at visit V1 or V2, and will verify that it was filled in correctly.

8 STUDY TREATMENTS

8.1 Treatments to be administered

All the patients in the study will continue therapy with IFNβ and with any drugs for flu-like syndrome (SOT), maintaining stable doses and frequency. These drugs will be dispensed to patients according to routine centre procedures.

All patients will take one capsule of cetirizine/placebo (hereinafter the "drug") one hour before the IFNβ injection. The drug will be packaged by the company Corden Pharma S.p.A., who will deliver it directly to the pharmacy of Tor Vergata University Hospital.

8.2 Identity of the study products

8.2.1 Packaging and labelling

Cetirizine, purchased from Farmacie Celesia S.r.l., is supplied as over-encapsulated tablets to be taken orally, in blister packs each containing 6 capsules. Each cetirizine tablet contains 10 mg of active ingredient.

The placebo is also encapsulated in capsules that are indistinguishable from those used for the cetirizine; these are also packaged in blister cards containing 6 tablets each.

The following shall appear on each blister pack:

Biogen Idec Italia S.r.l. - Study: BIIT 0212

Randomisation number

Batch number

First/Second month of treatment

Expiry date

The drug will be contained in two separate boxes to be given to the patient at the follow-up visits, each containing 3 blister cards for a total of 18 capsules of cetirizine or placebo, according to a randomisation list.

The following specifications will be shown on each box:

Biogen Idec Italia S.r.l. - Study: BIIT 0212

Randomisation number

First month of treatment or second month of treatment

Batch number

Expiry date

Store at a temperature below 30°C

"FOR CLINICAL TRIALS ONLY"

"Return the remaining capsules and used blister cards at the next follow-up visit"

"Keep out of the reach of children"

"Avoid release to the environment"

Lastly, the two boxes will in turn be placed into one box which will remain in the possession of the Investigator and which will be collected at the end of the study with the remaining drug to be disposed of.

The following specifications will be shown on these boxes:

Biogen Idec Italia S.r.l. - Study: BIIT 0212

Randomisation number

Batch number

Expiry date

Store at a temperature below 30°C.

"FOR CLINICAL TRIALS ONLY"

Each patient will be given a card to be kept with him/her at all times that shows: the name of the Investigator, the title of the study in which he/she is enrolled and the telephone number to call if needed.

The company appointed by Biogen Idec Italia S.r.l. to package, label and distribute Cetirizine to the Trial Centre is IBIS Informatica Srl, which has nominated Corden Pharma S.p.A., Viale dell'Industria, 3, Caponago (MB), which meets the legal requirements, and which will provide the certification to release the investigational drug in accordance with applicable laws, as the site responsible for preparing the boxes of drugs for patients and their logistics.

At the beginning of the study, the Investigator shall receive the drug needed for the treatment of 40 patients, plus 10 reserve patients, in a single delivery.

The drug must be stored at a temperature below 30°C.

8.3 Storage and delivery of the study drug

The drug will be stored in the pharmacy or in another safe place at the study centre, at a temperature below 30°C. The drug must be kept in its original packaging to protect it from direct light. One capsule of cetirizine/placebo will be administered one hour before the IFNβ injection.

At visit V1, the Investigator shall give each patient the box showing the patient's randomisation number and the words: FIRST MONTH, containing a sufficient number of capsules to cover the number of Interferon injections to be given at the highest administration frequency (14 injections/month), and shall explain the administration and storage method. At visit V2, the Investigator shall collect the unused drug and the used blister cards and give the patient the box coded with the same randomisation number and the words SECOND MONTH. When dispensing the drug packages, the Investigator shall note the date of delivery and quantity of drug dispensed in the medical record and the Case Report Form. In addition, the date and quantity of the drug provided to each patient must be recorded on an accounting form provided by the selected CRO. At visit V3 the patient must return the previously received packages to the Investigator, whether

used or not. These packages shall then be sent to the Sponsor who shall ensure that they are destroyed.

Each patient will be given a diary in which he/she must record the dates on which the drug was taken. The Investigator shall check compliance at the visits specified by the protocol.

The Investigator shall record the date the drug is returned by the patient and the number of unused capsules in the accounting form.

8.4 Randomisation procedure

When entering data into the centre's database, the patient will be automatically assigned a “Unique Code for Trial Subject (UCTS)” and a “Personal Identity Code (PIC)” which will clearly identify the case during the Study, at the same time as guaranteeing absolute anonymity and protection of the patient's identity. After verification of the inclusion/exclusion criteria (V1), the UCTS and PIC codes will be confirmed or, if the patient does not meet the criteria, they will be entered into the screening failure list. A randomisation number (RN) of 01 to 40 will be assigned at the time of randomisation, and linked to the PIC and will coincide with the number shown on the drug used by the patient.

During the study each patient will thus be identified by the Unique Code for Trial Subject (UCTS), the Personal Identification Code (PIC) and the RandCod (Randomisation Code, formed by the combination of PIC+RN), coinciding with the number on the assigned drug. The two codes (PIC+RN) will create the Randomisation Code, which will be shown on the questionnaire and patient diary.

Assignment of the UCTS, PIC, RN and RandCod is fully automated and does not require operator intervention.

This is a double-blind crossover study; all participating patients will therefore receive both cetirizine and the placebo according to a completely randomised sequence.

The randomisation list will cross over every 4 subjects and will be prepared by IBIS using the Nquery programme. This list will be held in a password protected PDF file, transferred to an electronic medium (CD/DVD/USB drive) and sent to the packaging manager of the Officina Farmaceutica Corden Pharma SpA by courier. The password to open the file will be sent separately. One copy of the list will be filed under the responsibility of the Scientific Director of IBIS Srl. Until the study is unblinded, only the Scientific Director of IBIS, the packaging manager for Corden Pharma SpA and their immediate colleagues may know the contents of the list.

Once the Sponsor authorizes the unblinding of the study, the list will be sent to both the IBIS biometry unit and to the Sponsor itself.

8.5 Blinding

This is a randomised, double-blind, crossover study. The investigators and the staff at the study centre, the Sponsor and the CRO will be blinded to the patient sequence for the entire duration of this Study. The Investigator will be given a closed and sealed envelope at the same time as the drug, containing the name of the drug (cetirizine or placebo) administered in the respective

sequence in order to be able to address any safety needs that may require the drug taken during the investigational therapy cycle to be known. The opening of this envelope must be authorized by Biogen Idec Italia Srl's Pharmacovigilance Unit and suitable justification provided in the e-CRF.

At the end of the research, all the open or still-sealed envelopes must be collected by the Clinical Monitor and filed in the Trial Master File (TMF).

8.6 Adherence to the treatment

At visits V2, V3 and in the event of a drop-out, Vx, the Investigator shall check the patient's compliance with treatment by examining the quantity of drug contained in the packages that the patient returns and the quantity reported in the diary.

Any discrepancy between the expected quantity and the quantity actually used shall be a measure of the patient's compliance with the prescribed posology; this information will be noted on the e-CRF.

8.7 Previous treatments and concomitant therapies

All treatment being received for RRMS must be documented in the e-CRF, including the current one.

Concomitant treatments for RRMS, such as symptomatic treatments, or treatments for other pathologies, may be used at the Investigator's discretion. Any changes to concomitant therapies must be reported in the e-CRF. If the patient is already taking drugs for the treatment of FLS, for example paracetamol or ibuprofen, considered as being the standard of therapy (SOT), they must continue to maintain the dose of that therapy as stable as possible during the study period, at the discretion of the Investigator and in accordance with standard clinical practice.

8.8 Special warnings and precautions for using the study drug

The elimination of Cetirizine may be compromised in patients with renal and liver failure. Caution should be taken in the administration of Cetirizine to these patients. Cetirizine may increase the effects of alcohol and depressants that act on the central nervous system. Therefore, caution is recommended in the assumption of alcohol and central nervous system depressants during the study.

Cetirizine may have a minimal or moderate influence on the patient's reactions. This must be taken into consideration when particular vigilance is needed, for example when driving.

The study product contains lactose monohydrate. Patients with rare hereditary galactose intolerance problems, Lapp lactase deficiency or glucose-galactose malabsorption should not take the medicinal product.

Data on a limited number of exposures to the drug during pregnancy suggest Cetirizine has no adverse effects on pregnancy or on the health of the foetus or newborn baby. Data on the excretion of Cetirizine in human milk are not available.

8.9 Side effects of the study drug

Taking Cetirizine may be associated with dry mouth, headache, dizziness, sleepiness, agitation, and abdominal and digestive disorders. Reversible disorders related to liver function, epileptic convulsions, thrombocytopenia, palpitations, tachycardia, syncope and weight gain have been observed. Rare cases of allergic reactions such as skin reactions and Quincke's edema have been reported.

9 PHARMACOVIGILANCE

9.1 Definitions

9.1.1 Adverse event

Adverse event (AE): any unfavourable medical event (signs, symptoms and abnormal laboratory results) occurring in a patient or in a subject participating in a clinical trial who has been given a pharmaceutical product, which does not necessarily have a causal relationship with the treatment.

AE intensity.

This is a subjective evaluation. The Investigator will use his or her own judgment to compare the adverse event with other similar events occurring in clinical practice. The intensity of any AE will be evaluated according to the following criteria:

- Mild: not causing limitation in normal activities. Subjects may complain of minor disorders.
- Moderate: causing certain limitations in normal activities. Subjects may complain of unpleasant disturbances.
- Severe: causing inability to carry out normal activities. Subjects may complain of unbearable disturbances or pain.

9.1.2 Serious adverse event

An SAE is any AE that:

- is fatal;
- poses a threat to life;
- requires hospitalisation or extends a current hospitalisation;
- leads to disability/continuing or significant incapacity;
- causes birth defects or congenital abnormalities;
- is a clinically significant medical event.

N.B.: the severity of an adverse event is not necessarily determined by its intensity. For instance, moderate nausea leading to hospitalisation shall be considered as serious.

9.2 Recording

Any data on adverse events (both serious and non-serious) occurring during the study must be collected and recorded in the e-CRF. It is essential to record the duration of any adverse event (onset and resolution date), its correlation with the drug, its outcome, and concomitant treatments, if any.

9.3 Correlation judgement

When making a judgement about correlation with the drug, the Investigator will consider the patient's clinical condition, concomitant therapies and the temporal relationship between the onset of the event and the administration of the investigational treatment. For every adverse event, the Investigator shall express a judgement on the possibility that the event can be plausibly correlated with the drug administered or not.

9.4 Adverse Drug Reaction (ADR)

Any harmful and unwanted response to a medicinal product occurring at a dose that is normally administered in humans for the prophylaxis, diagnosis or treatment of diseases, or to modify physiological functions.

The phrase “response to a medicinal product” means that the causal relationship between the AE and the medicinal product is at least a reasonable possibility, i.e. that such a relationship cannot be excluded. Therefore, in contrast to an AE, an ADR is characterised by the fact that a causal relationship between the AE and the medicinal product is considered at least possible.

9.5 Unexpected Adverse Drug Reaction

Any ADR whose nature and intensity do not correspond to the available information about the product (Summary of Product Characteristics or Investigator’s Brochure).

9.6 Notification of serious adverse events

The Investigator shall immediately report every SAE to the Biogen Idec pharmacovigilance manager and in any event within 24 hours of the event occurring or being made known to the Investigator by fax or e-mail using Biogen Idec's SAE form. Any event follow-up shall be reported to the Biogen Idec Pharmacovigilance manager within 24 hours.

The contact details of the Pharmacovigilance manager are the following:

Roberta Amodeo
Senior Associate I, Drug Safety
Biogen Idec Italia S.r.l.
Via Spadolini 5 – 20141 Milano
Tel. +39 02 58499043 - Mobile: +39 3425772172
Fax: +39 02 58499134
E-mail: farmacovigilanza@biogenidec.com

10 DATA MANAGEMENT AND STATISTICAL ANALYSIS

10.1 Data management

Study data will be recorded in the e-CRF, in electronic format, linked via the Internet to a central database. The Investigator will use the e-CRF in Italian to record patient data, collected by the patient in the original documentation. The Investigator will be able to access the e-CRF directly on the computer (after installation); no other technical support will be provided. The Investigator (and co-Investigators, where applicable) and the Clinical Monitor will have direct access to the e-CRF on the Investigator's computer using their own Login and password. The Clinical Monitor, the Sponsor and the Data Manager will also have access to the central database on the reference site using their individual Login and Password. Every access and/or modification to the local e-CRF and the central database will be tracked on a LOG file, available for consultation upon request. In order to ensure protection of the data recorded by each investigator, every user will be provided with individual access. Monitoring will be carried out by Clinical Trial Consulting ss staff on behalf of IBIS (CRO), designated by the Sponsor. Questionnaires will be checked by the CRO Monitor designated by the Sponsor during the scheduled monitoring visits at the centre, as an integral part of the centre's original documentation (e.g. clinical files, laboratory examination and radiology results etc.) who will also verify whether the Investigator has correctly entered the questionnaire and diary data into the e-CRF. When requests are made for support relating to entering data into the e-CRFs, the Investigator may contact the CRO at: Tel. 02/8330151; email: assistenza@ibisinformatica.com.

Compliance with applicable regulations on personal data protection will be ensured, in particular Legislative Decree no. 196 of 30 June 2003 and the Guidelines for the Guaranteeing of the protection of personal data – resolution no. 52 del 24/07/2008.

10.2 Sample size

The severity of the FLS expressed as the patient's judgement on a VAS ranging between 0 and 10 is the parameter used to calculate the sample.

The clinical hypothesis is that four hours after administering IFNβ, the average difference (d) of the VAS score between the two treatments is equal to or greater than 0.50, where d is expressed in standardized units.

Where $\mu_{Cetir.}$. is the mean VAS score obtained after administration of the standard therapy (SOT) plus cetirizine, and $\mu_{NoCetir}$ is the mean obtained after administration of the standard therapy alone (SOT), the difference between the two treatments will be expressed as:

$$d = \left| \mu_{noCetir.} - \mu_{Cetir.})/\sigma \right| = 0.50$$

where d is the effect size expressed in standard units and σ is the standard deviation of the difference.

Therefore the null hypothesis (two tailed) is

H0: $|\mu_{noCetir.} - \mu_{Cetir.})/\sigma| \leq 0.50$ - or d=0;

While the alternative hypothesis is

Ha: $|\mu_{noCetir.} - \mu_{Cetir.})/\sigma| > 0.50$ - or d≠0.

Selecting * = 0.05 and β = 0.20 (power =80%), it is necessary to enrol 34 patients, who will be sequentially treated in accordance with a crossover design, as shown in the randomisation list.

Taking into consideration the possibility of a 15-20% drop-out rate, the sample to be randomised for the two sequences will be made up of 40 subjects.

10.3 Statistical analysis

10.3.1 Descriptive analysis

All the variables recorded in the Case Report Form will be reported in descriptive tables. In particular, continuous variables will be reported as a mean and SD, while nominal or discrete variables will be summarised in contingency tables.

10.3.2 Inferential statistics

The critical threshold of statistical significance for the tests shown below is P = 0.05.

10.3.3 Uniformity of the sequences to the baseline

To evaluate the uniformity of the two sequences, continuous parameters will be evaluated using the Student's t test for independent data, while the nominal or discrete parameters will be evaluated using the chi-square test with the continuity correction in the event of 2x2 tables.

10.3.4 Efficacy

The efficacy of Cetirizine in reducing FLS symptoms in patients with RRMS being treated with IFNβ will be evaluated 4 hours after IFNβ administration (after 4 weeks of therapy within each sequence)

The VAS and FLS-S score variables will be analysed using analysis of variance (ANOVA) with repeated measurements. In accordance with the crossover design of the study, the carry-over effect and the sequence effect will be evaluated in addition to the treatment effect.

During the treatment, the severity of the FLS, evaluated using both the VAS and FLS-S scores and recorded 4 and 12 hours after the injection, will be analysed using analysis of variance with repeated measurements. In accordance with the crossover design of the study, the carry-over effect and the sequence effect will be evaluated in addition to the treatment effect.

Moreover, appropriate multiple comparisons will be made to evaluate the time of the treatment between and within groups.

The same analysis in line with the crossover design will be performed on the values of the area under the curve (AUC) of the mean VAS and FLS-S values, recorded 4 and 12 hours after the injection for each treatment.

Lastly, the severity scores for flu-like syndrome (FLS-S) will be compared with the baseline scores. The patients for whom the score is increased by 2 or more units will be defined as positive for FLS and their frequencies will be analysed using the Armitage method, according to the crossover design.

10.3.5 Compliance

Evaluation of compliance variations will be performed in cases where the therapy is changed.

10.3.6 Adverse events

Adverse event frequency will be analysed descriptively. In particular, we will analyse the information about treatment compliance and Flu-like syndrome recorded in the patient diary

10.3.7 Data recording

Study data will be recorded in the e-CRFs linked via the Internet to a central database. Anonymous and encrypted data is sent through a personalised access key, which is different for each participating Investigator (according to the provisions of the Code on protection of personal data, Attachment B. Technical regulations on minimum security measures).

All anonymous data recorded electronically may be accessed but not modified by staff designated by the CRO and/or Sponsor, who will access the e-CRFs via a separate personal account.

After being entered in the central database, any changes to the database will be made using a query issue and resolution system on the project's website. Data in the e-CRFs are saved locally in a confidential format on the computer provided by the Centre. After collecting the data, the Investigator can transfer the electronic file to a CD which will be kept in the Trial Master File.

Compliance with applicable regulations on personal data protection will be ensured, in particular Legislative Decree no. 196 of 30 June 2003 and the Guidelines for the Guaranteeing of the protection of personal data – resolution no. 52 del 24/07/2008.

10.3.8 Criteria for evaluation of data

In regards to efficacy, all randomised patients who have taken at least one dose of antihistamine and of placebo and who have had at least one pre and post drug evaluation for both products will be evaluated, in accordance with the crossover design Intention-To-Treat. Patients who do not meet these four requirements may not be evaluated for efficacy.

On the other hand, those patients who took at least one dose of antihistamine or one of placebo will be evaluated for safety.

10.3.9 Withdrawal of patients from the study

Patients shall have the right to withdraw from the study at any time, with no obligation to give a reason, and without prejudice to clinical management according to standard practice. Patients who withdraw will be requested to attend a final visit at the centre in order to collect data about their clinical condition, and to collect their diary. However, patients have the right to refuse to do so.

11 ACCESS TO ORIGINAL DATA/DOCUMENTS

The Investigator acknowledges that the Sponsor, its employees or designated staff have the right to verify and analyse the clinical documentation about this Study throughout its duration.

The Investigator shall also permit inspections by the competent Ethics Committee and/or Competent Authorities.

12 MONITORING

Monitoring will be carried out by Clinical Trial Consulting ss staff on behalf of IBIS (CRO), designated by the Sponsor. In general, visits to the centre are scheduled for before the commencement of the Study, during the Study and on terminating the Study. Monitoring visits will check for the completeness of data recorded in the e-CRFs and their compliance with the protocol throughout the duration of the Study.

Questionnaires will be checked by the CRO Monitor designated by the Sponsor during the monitoring visits scheduled at the centre, as an integral part of the centre's original documentation (e.g. clinical files, laboratory and radiology examination results, etc.) who will also verify whether the Investigator has correctly entered the questionnaire and diary data into the e-CRF.

13 AMENDMENTS

If necessary, this protocol may be amended. All amendments shall be numbered and dated, and authorised by all signatories of the Protocol, reported and/or approved by the Ethics Committee prior to their implementation, unless they refer to logistic or administrative aspects of the Study. Reporting and/or approval of the Study shall take place in accordance with the provisions of the Ministerial Decree of 21/12/2007, published in the Ordinary Supplement of OGIR no. 53 of 3 March 2008.

14 ETHICS/REGULATORY REQUESTS

This Study will be conducted in compliance with all protocol provisions, and current national and EU provisions regulating clinical trials, and in compliance with the ethical and professional principles of medical practice (Legislative Decree of 24 June 2003 no. 211 implementing the EU Directive on the application of good clinical practice in executing clinical drug trials, the Declaration of Helsinki, and the Oviedo Convention on Human Rights and Biomedicine, Legislative Decree 200/2007, etc.

14.1 Study authorisation

All documentation relating to amendments to the Study Protocol, adverse events and suspension of the Study must be sent to the Ethics Committee exclusively via the CRO.

All expenses connected with submission of the documents and the approval procedures or the presentation of Protocol amendments to the Ethics Committee will be at the exclusive expense of the CRO.

The study centre will not alter or modify the Protocol nor permit the PRINCIPAL INVESTIGATOR or any member of the staff to alter or modify the Protocol in any way without the express written consent of Biogen Idec Italia and the approval of the competent Ethics Committee, unless required by law for the purpose of protecting the health, safety or rights of the Subjects.

According to the provisions of Legislative Decree 211 of 24 June 2003 (Official Gazette No. 184 of 9 August 2003), the Protocol must be submitted to the Ethics Committee which, in the case of monocentric trials, must communicate its reasoned opinion to the sponsor, the Minister of Health and the competent authorities within 60 days of receiving the application presented by Biogen Idec Italia.

During the period in which the application is being examined, the Ethics Committee may submit a one-time-only request for additional information to Biogen Idec.

The Study may commence at the research centre only upon written approval by the Ethics Committee, signing of the financial agreement with the centre, and issuance of the relevant Administrative Resolution.

15 INFORMED CONSENT FORM AND INFORMATION LEAFLET

Enrolment may only begin after completion of the authorisation procedure and commencement of the Study at the centre, and following the opening visit by the designated Monitor. Patients shall be provided with all available information about the Study included in the Information Leaflet attached to the Informed Consent Form, whose version must be approved by the Ethics Committee of the centre in which the subject is enrolled.

When consenting to participate in the study, all subjects selected to participate must confirm their agreement by signing and dating the Informed Consent Form in person and receive a copy of it, at the time of joining the Study. The original copy of the Informed Consent Form shall be filed by the Investigator in the Investigator Site Folder, which is part of the TMF and which the Investigator is obliged to retain.

All subjects participating in the Study shall be informed that their personal data may be accessed by Biogen Idec staff and/or the staff of the Company designated by the competent authorities in a confidential manner, according to applicable laws and regulations (Guidelines for the protection of personal data - Resolution no. 52 of 24/07/2008).

All documents that may lead to the identification of subjects will be kept strictly confidential, and made publicly available only in accordance with applicable laws and/or regulations. If the study results are published, the identity of each subject will remain anonymous. Patients shall be informed that participation in the Study is voluntary, that refusal to participate does not entail any

penalty and that they have the right to withdraw from the Study at any time, without prejudice to the right to receive future health care.

Furthermore, patients shall authorise the use of their data in accordance with Legislative Decree no. 196 of 30 June 2003 (Personal Data Protection Code).

Patient data will be clearly recorded in the related e-CRFs by the Investigator, but clinical data will be exported in an anonymous format. Therefore, identification of patients outside the centre will be possible only by using the code assigned to each patient.

However, the Investigator shall keep a list containing the names of subjects and the identifying information mentioned above in the Investigator Site Folder retained at the centre. This list shall remain in the trial centre even after the completion of the Study; on no condition shall this list be sent to the Sponsor and/or the Companies (CROs) designated by the Sponsor.

16 STUDY DISCONTINUATION

Early discontinuation of the Study shall be reported in writing to the Regulatory Authorities and to the Principal Investigator. In the event of discontinuation, the communication procedures between the Sponsor and/or the Companies charged with the task of managing the Study in the name and on behalf of the Sponsor and the centre will be detailed in the Agreement signed by the Parties involved.

17 FINAL REPORT

Upon completion of the Study, the Sponsor will prepare a final report outlining the Study objectives, methodology, clinical observations, and results in relation to the Study objectives, based on the statistical results obtained.

18 DOCUMENT STORAGE

All documents containing the original data required by the Protocol and recorded in the e-CRFs shall remain at the centre participating in the Study.

Pursuant to applicable regulations implemented by the centre and/or applicable law on data retention, the Investigator is required to retain a copy of the Protocol, the final report, and the Case Report Forms, along with the original Informed Consent Forms and documentation regarding the Study, for at least 2 years. If an individual research centre requests a different retention period, this period shall always be specified in the financial agreement with the trial centre. The Investigator cannot destroy any saved document relating to the Study without prior written approval from the Sponsor.

19 INSURANCE COVERAGE

For the Subjects involved in the Study, Biogen Idec Italia has taken out Insurance Policy no. 390-01583722-14022 with the HDI Gerling Insurance company for third-party liability, providing risk coverage for any damages arising from participation in the Study pursuant to the Decree of 14 July 2009 on “Minimum requirements for insurance policies to protect Subjects participating in

clinical trials of medicinal products". The Investigator will be given a copy of the insurance policy before the study begins and will also receive any updates during the study.

20 FINANCIAL TERMS AND CONDITIONS

Cetirizine, the drug used in this study, will be provided free of charge by Biogen Idec Italia. The costs of the required medical visits shall be borne by Biogen Idec Italia.

All financial terms and conditions regulating this Study shall be included in an Agreement made between the trial centre and the Sponsor, or Companies designated by them, acting in the name of and on behalf of Biogen Idec Italia.

21 USE OF INFORMATION AND PUBLICATION OF DATA

All results, information and findings of which the Investigator may become aware, either directly or indirectly, throughout the duration of the Study, or deriving from the Study, including any industrial or intellectual copyright, shall be the sole property of the Sponsor.

Pursuant to the "regulations on data transparency and publication" contained in Ministry of Health Circular no. 6 of 2 September 2002, published in OGIR no. 214 of 12 September 2002, and Article no. 5, section c) of the Ministerial Decree of 12 May 2006, published in the Official Gazette no. 194 of 22 August 2006, publication of the data obtained upon completion of the Study is subject to no restrictions. Therefore, they may be made available to the scientific community at the discretion of the Investigator who has conducted the Study, subject to prior notification to the Sponsor, in order to verify the confidentiality, completeness and truthfulness of the information and the correctness of the results.

22 REFERENCES

Brandes DW, Bigley K, Hornstein W, Cohen H, Au W, Shubin R. (2007) "Alleviating flu-like symptoms with dose titration and analgesics in MS patients on intramuscular interferon beta-1a therapy: a pilot study." Curr Med Res Opin 23(7): 1667-1672.

EMA. (2008) Retrieved 02/10/2012, 2012, from http://www.ema.europa.eu/docs/it_IT/document_library/Referrals_document/Zyrtec_30/WC500008284.pdf.

Galetta S.L and Markowitz C. (2005) "US FDA-approved disease-modifying treatments for multiple sclerosis: review of adverse effect profiles." CNS Drugs 19(3): 239-252.

Galetta SL, Markowitz C, Lee AG (2002). "Immunomodulatory agents for the treatment of relapsing multiple sclerosis: a systematic review." Arch Intern Med 162(19): 2161-2169.

Johns MW (1991). "A new method for measuring daytime sleepiness: the Epworth sleepiness scale." Sleep 14(6): 540-545.

Marone G, Gentile M, Petraroli A, De Rosa N, Triggiani M. (2001) "Histamine-induced activation of human lung macrophages." Int Arch Allergy Immunol 124(1-3): 249-252.

Marone G, Granata F, Spadaro G, Onorati AM, Triggiani M. (1999) "Antiinflammatory effects of oxatomide." J Investig Allergol Clin Immunol 9(4): 207-214.

Martinez-Caceres EM, Rio J, Barrau M, Duran I, Borras C, Tintore M, Montalban X. (1998). "Amelioration of flulike symptoms at the onset of interferon beta-1b therapy in multiple sclerosis by low-dose oral steroids is related to a decrease in interleukin-6 induction." Ann Neurol 44(4): 682-685.

Mohr DC, Likosky W, Boudewyn AC, Marietta P, Dwyer P, Van der Wende J, Goodkin DE. (1998) "Side effect profile and adherence to in the treatment of multiple sclerosis with interferon beta-1a." Mult Scler 4(6): 487-489.

Montalban X, Duran I, Rio J, Saez-Torres I, Tintore M, Martinez-Caceres EM. (2000) "Can we predict flu-like symptoms in patients with multiple sclerosis treated with interferon-beta?" J Neurol 247(4): 259-262.

Reess J, Haas J, Gabriel K, Fuhlrott A, Fiola M. (2002) "Both paracetamol and ibuprofen are equally effective in managing flu-like symptoms in relapsing-remitting multiple sclerosis patients during interferon beta-1a (AVONEX) therapy." Mult Scler 8(1): 15-18.

Rice GP, Ebers GC, Lublin FD, Knobler RL. (1999) "Ibuprofen treatment versus gradual introduction of interferon beta-1b in patients with MS." Neurology 52(9): 1893-1895.

Rio J, Nos C, Bonaventura I, Arroyo R, Genis D, Sureda B, Ara JR, Brieva L, Martin J, Saiz A, Sanchez Lopez F, Prieto JM, Roquer J, Dorado JF, Montalban X. (2004) "Corticosteroids, ibuprofen, and acetaminophen for IFNbeta-1a flu symptoms in MS: a randomised trial." Neurology 63(3): 525-528.

Rio J, Nos C, Marzo ME, Tintore M, Montalban X. (1998) "Low-dose steroids reduce flu-like symptoms at the initiation of IFNbeta-1b in relapsing-remitting MS." Neurology 50(6): 1910-1912.

Roth J, Aslan T, Storr B, Zeisberger EB. (1997). "Lack of cross tolerance between LPS and muramyl dipeptide in induction of circulating TNF-alpha and IL-6 in guinea pigs." Am J Physiol 273(4 Pt 2): R1529-1533.

Tremlett HL and Oger J. (2003) "Interrupted therapy: stopping and switching of the beta-interferons prescribed for MS." Neurology 61(4): 551-554.

Triggiani M, Gentile M, Secondo A, Granata F, Oriente A, Taglialatela M, Annunziato L, Marone G. (2001) "Histamine induces exocytosis and IL-6 production from human lung macrophages through interaction with H1 receptors." J Immunol 166(6): 4083-4091.

Vignatelli L, Plazzi G, Barbato A, Ferini-Strambi L, Manni R, Pompei F, D'Alessandro R, on behalf of GINSEN* (2003). "Italian version of the Epworth sleepiness scale: external validity". Neurol Sci 23:295–300.

Statistical references

Machin, David and Campbell, Michael J. (1987) Statistical Tables for the Design of Clinical Trials. Blackwell Scientific Publications, Boston.

Cohen J. (1988) Statistical Power Analysis for the Behavioural Sciences, Lawrence Erbaum Associates, Hillsdale, New Jersey.

Steel RGD, Torrie JH, Dickey DA. (1997) Principles and Procedures of Statistics – A Biometrical Approach. Third Edition, McGraw-Hill International Book Company, London

Armitage P, Berry G, Matthews JNS. (2002) Statistical methods in Medical Research. 4rd Edition, Blakwell [sic: Blackwell] Scientific Publications, Oxford, England.

Siegel S, Castellan NJ Jr. (1992) Statistica non Parametrica 2a Edizione, McGraw-Hill Libri Italia – Milano

Hsieh FY. Sample Size Tables for Logistic Regression, Statistics in Medicine, Vol. 8, 795-802 (1989)
